# MRI Signatures of Parotid Tumours Impacting Management Decisions: A Retrospective Study With Radiology and Pathology Correlation

**DOI:** 10.1111/1754-9485.13865

**Published:** 2025-05-19

**Authors:** Nivedita Chakrabarty, Prathamesh Pai, Arpita Sahu, Oindrila Roy Chowdhury, Pashmina Kandalgaonkar, Tapish Dadlani, Munita Menon, Suman Kumar Ankathi

**Affiliations:** ^1^ Department of Radiodiagnosis Advanced Centre for Treatment, Research and Education in Cancer (ACTREC), Tata Memorial Centre, Homi Bhabha National Institute (HBNI) Parel, Mumbai Maharashtra India; ^2^ Department of Surgical Oncology Tata Memorial Hospital, Tata Memorial Centre, Homi Bhabha National Institute (HBNI) Parel Mumbai Maharashtra India; ^3^ Department of Radiodiagnosis Tata Memorial Hospital, Tata Memorial Centre, Homi Bhabha National Institute (HBNI) Parel, Mumbai Maharashtra India; ^4^ Department of CRS Tata Memorial Hospital Mumbai Maharashtra India; ^5^ Department of Pathology Tata Memorial Hospital, Tata Memorial Centre, Homi Bhabha National Institute (HBNI) Parel, Mumbai Maharashtra India

**Keywords:** benign and malignant parotid tumours, grades of mucoepidermoid carcinoma, MRI of parotid tumours

## Abstract

**Introduction:**

Fine needle aspiration (FNA) from parotid tumour is inadequate and nondiagnostic in 8% and FNA/biopsy from deep lobe is technically challenging; hence, our first objective was to evaluate MRI findings which best predict the benign and malignant nature of parotid tumour. Our second objective was to develop MRI signatures for parotid tumour histopathologies including grades of carcinoma, to help in decision making regarding elective neck dissection.

**Methods:**

Two head and neck radiologists retrospectively evaluated and developed signatures of common benign and malignant parotid tumours using morphology and signal intensity–related variables for 98 patients on MRI available in PACS from 01 January 2016 to 26 December 2022. T1 weighted image (WI), T2WI, short tau inversion recovery, diffusion WI/apparent diffusion coefficient and postcontrast T1WI sequences were evaluated. The developed MRI signatures were then validated by a blinded third radiologist.

**Results:**

Sensitivity, specificity, accuracy, positive and negative predictive values using MRI signatures were 92.31%, 100%, 94.23%, 100% and 81.25%, respectively, for benign and malignant nature of parotid tumours with a highly significant *p*‐value (< 1e‐04). Developed MRI signatures also showed high statistical performance and significant *p‐*value for parotid tumour histopathologies and grades of mucoepidermoid carcinoma (MEC). T2 signal intensity and enhancement patterns can help identify low‐grade MEC, impacting management decisions regarding elective neck dissection.

**Conclusions:**

MRI can predict the benign and malignant nature, parotid tumour histopathologies and grades of MEC when typical signatures are present, impacting management decisions.

## 1 Introduction

Salivary gland tumours constitute approximately 2%–6.5% of all head and neck cancers and 0.5% of all malignancies [1]. Seventy per cent of salivary gland tumours are located in the parotid gland, of which 80% are benign [1, 2]. Pleomorphic adenoma (PA) is the commonest benign parotid gland tumour (65%) followed by Warthin tumour (WT) [15%–20%]. Mucoepidermoid carcinoma (MEC) [30% of malignant tumours] is the commonest malignant parotid tumour [3]. Imaging plays a key role in detecting, localising and characterising parotid tumours and for presurgical planning. Ultrasound is the initial imaging modality for evaluating parotid tumours located in the superficial lobe; however, it has limitations for evaluating deeper structures. Magnetic resonance imaging (MRI) is the preferred modality for parotid tumour delineation and characterisation, including those located in the deep lobe. In addition, MRI is the modality of choice for evaluating perineural spread, intracranial extension and extension to the skull base [2, 4].

Precise diagnosis of WT on imaging is essential as a conservative nonsurgical approach and active surveillance may be suggested for willing patients as they do not undergo malignant transformation [5, 6, 7]. Fine needle aspiration (FNA) is inadequate and nondiagnostic in 8% of cases, and if MRI findings are highly suggestive of WT, then further invasive core needle biopsy can be obviated [4, 6]. Moreover, it has been found that FNA is less accurate for differentiating WT from malignant parotid tumours [8], requiring MRI for diagnosis to obviate biopsy.

Low‐grade primary parotid malignancies do not require elective neck dissection (END) for clinically negative neck nodes, whereas END is recommended in high‐grade parotid malignancies [4, 9]. FNA/biopsy from the deep lobe can be technically difficult, further complicating inadequate FNA.

The objectives of our study were to evaluate the MRI findings which best predict the benign and malignant nature of parotid tumours, and to assess whether MRI signatures could be assigned to parotid tumour histopathologies, including different grades of carcinoma.

## 2 Methods

### 2.1 Study Population

All patients with histopathologically confirmed parotid tumour were retrospectively evaluated in a tertiary cancer hospital from 01 January 2016 to 26 December 2022, after obtaining Institutional Ethical Committee clearance. Ninety eight patients who fit inclusion and exclusion criteria were enrolled. Both male and female of all age groups were retrospectively analysed.

### 2.2 Inclusion Criteria

1. Patients with biopsy/FNA proven parotid tumours whose baseline MRI was available on picture archiving and communication system (PACS).
2. MRI sequences required for evaluation: T1 weighted imaging (WI), T2WI, short tau inversion recovery (STIR), diffusion WI (DWI)/apparent diffusion coefficient (ADC), postcontrast T1WI.

### 2.3 Exclusion Criteria

1. Patients whose baseline MRI was not available on PACS.
2. Poor quality scans with inadequate sequences.

### 2.4 Imaging Analysis

Two radiologists (Radiologists 1 and 2), having more than 10‐year experience in head and neck oncoimaging, independently evaluated MRI images available on PACS for all 98 patients. MRI images were acquired either in General Electric (GE)‐Signa explorer 1.5T or Philips‐Ingenia 1.5T, and the sequences evaluated were: T1WI, T2WI, STIR, DWI/ADC, postcontrast T1WI. Echo‐planar imaging technique was used for DWI with b‐value 800. Parameters/variables evaluated were: laterality (unilateral/bilateral), number of tumours, location of tumour within parotid gland (superficial lobe/deep lobe/both superficial and deep lobes/parotid tail), size (≤ 2 cm/> 2– ≤ 4 cm/> 4 cm), shape (oval/round/irregular), margins (well‐defined smooth/well‐defined lobulated/ill‐defined/infiltrative/part‐infiltrative, part‐lobulated), capsule (present/absent/present, interrupted), along with the following MRI signal characteristics:

- Any T1 hyperintense component within the tumour (irrespective of the size of component): present/absent.
- Fat component within the tumour (tumour having any visible T1 hyperintense component which was clearly suppressed on STIR, was considered to have a fat component irrespective of the size of fat component): present/absent.
- Predominant T2 hyperintensity in the solid component (hyperintense to parotid gland): present/absent.
- Any T2 hypointensity (hypointense to parotid gland, which may be hyperintense or isointense to muscle) within the solid component of the tumour: present/absent.
- Any T2 hypointensity which is isointense to muscle, within the solid component: present/absent.
- Presence of cystic component within the tumour: yes/yes, with papillary projections/no.
- Diffusion restriction within the tumour: absent/patchy (heterogeneous diffusion restriction throughout the tumour)/homogeneous (entire tumour showing uniform diffusion restriction)/part of the lesion (solid nodule within a cyst showing diffusion restriction).
- Contrast enhancement of the solid component within tumour: homogeneous/heterogeneous/hypoenhancing (enhancing less than the parotid gland)/rim enhancement.
- Extraglandular extension of tumour: present/absent. Structures involved (if present).
- Metastatic cervical lymphadenopathy: present/absent/indeterminate.

Both Radiologists 1 and 2 were nonblinded and developed MRI signatures for parotid tumours, and any discrepancies between them were mutually resolved.

### 2.5 Histopathological Correlation

Correlation of extraglandular extension on MRI was done with postoperative histopathological report (HPR) [for those patients who were operated and had this information documented in the post operative HPR]. Metastatic cervical lymph nodes observed on MRI were also correlated with postoperative histopathological report (HPR)/fine needle aspiration (FNA) report.

### 2.6 Validation

Radiologist 3 (with 5‐year experience in oncoimaging) was blinded to the HPR of the same 98 patients and predicted the benign and malignant nature, parotid tumour histopathologies and grades of MEC using the developed MRI signatures by Radiologists 1 and 2.

### 2.7 Statistical Analysis

Statistical analysis has been performed using SPSS (the statistical package for social sciences) IBM Corp. Released 2017. IBM SPSS Statistics for Windows, Version 25.0. Armonk, NY: IBM Corp and R and R Studio 4.1 version. Descriptive analysis was used to summarise data. Continuous variables were presented as mean ± standard deviation (SD) or median (interquartile range) based on the normality of data. Categorical data were presented as numbers (percentages). The normality of data was assessed using the Shapiro–Wilk/Kolmogorov test depending upon sample size. All analyses were two‐sided, and significance was set at a *p*‐value of < 0.05. The chi‐square test was used to obtain the association between categorical variables. Developed MRI signatures were validated using *p*‐value, sensitivity, specificity, accuracy, positive predictive value (PPV) and negative predictive value (NPV).

## 3 Results

### 3.1 Patient Characteristics

The mean age and frequency of males and females in the total population, and amongst benign and malignant parotid tumours are shown in Table S1 (in supplementary file 4). The mean age of female patients (34) was 47.4 years ± 16.2 SD and the mean age of male patients (64) was 51.7 years ± 16.5 SD. Overall mean age was 50.2 years + 16.5 SD.

### 3.2 Surgical Characteristics

Eighty patients were operated; 27 patients underwent radical parotidectomy, 24 patients underwent superficial parotidectomy, 18 patients underwent conservative parotidectomy, 4 patients underwent adequate parotidectomy, 3 patients underwent extended radical parotidectomy, 1 patient underwent extracapsular dissection, 1 patient underwent right extracapsular dissection and left superficial parotidectomy, 1 patient underwent bilateral adequate parotidectomy and 1 patient underwent left superficial parotidectomy and right adequate parotidectomy.

Sixty‐six patients underwent neck dissection and one patient got FNA of cervical nodes.

### 3.3 Histopathological Correlation

There was a significant correlation between the radiological diagnosis of extraglandular extension and postoperative HPR, and between metastatic cervical nodes and postoperative HPR/FNA.

### 3.4 Benign versus Malignant Parotid Tumours

Out of 98 patients, 26 had benign and 72 had malignant tumours. PA was the most common benign histopathology and MEC was the most common malignant histopathology. Three patients in our study had tumours in both parotid glands and all were WT. Hence, a total of 104 tumours were evaluated, out of which 32 were benign and 72 were malignant. Tumour‐wise analysis was performed for morphology and MRI signal intensity–related variables, except for metastatic cervical nodes and laterality of tumours, for which patient‐wise analysis was performed.

Tables S2 and S3 (in supplementary file 4) show various benign and malignant histopathologies, respectively, with the number of lesions in each category.

### 3.5 Morphology‐Related Variables Evaluated on MRI

There was a significant difference (*p* value < 0.05) between benign and malignant tumours for the following morphological variables: laterality, location within the parotid gland, size, capsule, margins, shape, extraglandular extension and metastatic cervical nodes.

Majority of the benign tumours were of oval shape (43.8%), followed by round shape (37.5%), and showed well‐defined lobulated margins in the majority (53.1%) followed by well‐defined smooth margins (34.4%). WT was the only tumour that was bilateral. Multiple tumours in the same parotid gland were also WT. Most of the benign tumours were completely encapsulated (81.2%). None of the benign tumours had infiltrative margins. Most of the malignant tumours were irregular (70.8%) in shape, showed infiltrative margins throughout (50.0%) followed by well‐defined lobulated margins (27.8%). Size of tumour > 4 cm was seen in 40.3% of the malignant and only 9.4% of the benign tumours, whereas size ≤ 2 cm was seen in 34.4% of the benign and 11.1% of the malignant tumours. Tumours occupying both superficial and deep lobes were found in 41.7% of the malignant tumours and 15.6% of the benign tumours.

Three of the benign tumours showed extraglandular extension; two were PA which showed extension to the parapharyngeal space from the deep lobe, and one was a large encapsulated lipoblastoma which showed extraglandular extension merely because of its size. All three tumours had smooth margins without any infiltration to adjacent organs.

### 3.6 Signal Intensity–Related Variables Evaluated on MRI

There was a significant difference (*p*‐value < 0.05) between benign and malignant tumours for the following signal intensity–related variables: any visible T1 hyperintensity within the tumour (with/without fat), presence of fat within the tumour, T2 hypointensity within the solid component of tumour isointense to muscle signal, predominant T2 hyperintensity in the solid component and type of contrast enhancement.

Majority of the benign tumours showed T1 hyperintensity [with/without fat content] (68.8%). Although 59.4% of the benign tumours showed T2 hypointense signal intensity compared to the parotid gland, none of them showed T2 hypointense signal which was isointense to muscle. On the other hand, 18.1% of the malignant tumours showed T2 hypointensity isointense to muscle. Fat component was present in 37.5% of the benign tumours, whereas 12.2% of the malignant tumours showed fat content. T1 hyperintensity was absent in the majority (56.9%) of the malignant tumours. Twenty‐five per cent of the benign tumours were hypoenhancing while only 9.7% of the malignant tumours showed hypoenhancement, and 25% of the malignant tumours showed homogeneous enhancement while only 6.2% of the benign tumours showed homogeneous enhancement.

Table 1 shows lesion‐wise analysis of MRI variables and their level of significance between benign and malignant tumours, and Table 2 shows patient‐wise analysis of laterality and metastatic cervical nodes and their level of significance between benign and malignant tumours.

**TABLE 1:** Lesion‐wise analysis of MRI variables and their level of significance between benign and malignant tumours.

Variable	Benign (*n* = 32)	Malignant (*n* = 72)	Total (*n* = 104)	*p*
**Location**				
Both	5 (15.6)	30 (41.7)	35 (33.7)	
Deep	2 (6.2)	3 (4.2)	5 (4.8)	
Superficial	17 (53.1)	36 (50)	53 (51.0)	
Superficial, parotid tail	8 (25.0)	3 (4.2)	11 (10.6)	0.003194
**Shape**				
Irregular	6 (18.8)	51 (70.8)	57 (54.8)	
Oval	14 (43.8)	8 (11.1)	22 (21.2)	
Round	12 (37.5)	13 (18.1)	25 (24.0)	< 1e‐04
**Size**				
> 4	3 (9.4)	29 (40.3)	32 (30.8)	
> 2 to ≤ 4	18 (56.2)	35 (48.6)	53 (51.0)	
≤ 2	11 (34.4)	8 (11.1)	19 (18.3)	0.001063
**Margins**				
Infiltrative	0 (0.0)	36 (50)	36 (34.6)	
Ill‐defined	4 (12.5)	4 (5.6)	8 (7.7)	
Well‐defined lobulated	17 (53.1)	20 (27.8)	37 (35.6)	
Well‐defined smooth	11 (34.4)	7 (9.7)	18 (17.3)	
Part infiltrative, part lobulated	0 (0.0)	5 (6.9)	5 (4.8)	< 1e‐04
**Capsule**				
Absent	1 (3.1)	33 (45.8)	34 (32.7)	
Present	26 (81.2)	17 (23.6)	43 (41.3)	
Present, interrupted	5 (15.6)	22 (30.6)	27 (26.0)	< 1e‐04
**Any T1 hyperintensity**				
Present	22 (68.8)	31 (43.1)	53 (51.0)	
Absent	10 (31.2)	41 (56.9)	51 (49.0)	0.027334
**Fat**				
Absent	20 (62.5)	63 (87.5)	83 (79.8)	
Present	12 (37.5)	9 (12.5)	21 (20.2)	0.007662
**Any T2 hypointense signal to gland**				
Present	19 (59.4)	52 (72.2)	71 (68.3)	
Absent	13 (40.6)	20 (27.8)	33 (31.7)	0.284181
**Any T2 hypointensity isointense to muscle**				
Absent	32 (100.0)	59 (81.9)	91 (87.5)	
Present	0 (0.0)	13 (18.1)	13 (12.5)	0.024547
**Presence of cystic component**				
Yes	8 (25.0)	30 (41.7)	38 (36.5)	
No	24 (75.0)	38 (52.2)	62 (59.6)	
Yes, with papillary projections	0 (0.0)	4 (5.6)	4 (3.8)	0.070753
**Pattern of diffusion restriction**				
Patchy	15 (46.9)	31 (43.1)	46 (44.2)	
Part of the lesion	1 (3.1)	12 (16.7)	13 (12.5)	
Homogeneous	5 (15.6)	16 (22.2)	21 (20.2)	
Absent	11 (34.4)	13 (18.1)	24 (23.1)	0.095464
**Pattern of contrast enhancement of the solid component**				
Heterogeneous	19 (59.4)	43 (59.7)	62 (59.6)	
Homogeneous	2 (6.2)	18 (25)	20 (19.2)	
Hypoenhancing	8 (25.0)	7 (9.7)	15 (14.4)	
Rim enhancement	3 (9.4)	4 (5.6)	7 (6.7)	0.043678
**Predominant T2 hyperintensity in the solid component**				
Present	15 (46.9)	12 (16.7)	27 (26.0)	
Absent	17 (53.1)	62 (83.3)	77 (74.0)	0.002693
**Extraglandular extension**				
Present	3 (9.4)	41 (56.9)	44 (42.3)	
Absent	29 (90.6)	31 (43.1)	60 (57.7)	< 1e‐04

**TABLE 2:** Patient‐wise analysis of metastatic cervical nodes and laterality of tumour, and their level of significance between benign and malignant tumours.

Variable	Level	Malignant (*n* = 72)	Benign (*n* = 26)	Total (*n* = 98)	*p*
Metastatic cervical lymph nodes	Absent	45 (62.5)	25 (96.2)	70 (71.4)	0.004914
	Present	23 (31.9)	1 (3.8)	24 (24.5)	
	Indeterminate	4 (5.6)	0 (0.0)	4 (4.1)	
Laterality	Right	32 (44.4)	9 (34.6)	41 (41.8)	0.012532
	Left	40 (55.6)	14 (53.8)	54 (55.1)	
	Bilateral	0 (0.0)	3 (11.5)	3 (3.1)	

Based on these findings, an oval or round‐shaped, completely encapsulated tumour with well‐defined smooth margins containing fat is more likely to be benign, provided extraglandular extension and metastatic cervical nodes are absent. Bilaterality increases the likelihood of benignity.

Any parotid tumour showing infiltrative margins, and/or extraglandular extension with infiltration into surrounding structures, and/or metastatic cervical nodes, and/or T2 hypointense component isointense to muscle, and/or cyst with papillary projections, is malignant. Location of tumour in both superficial and deep lobes, absence of predominant T2 hyperintensity in the solid component and homogeneous enhancement increase the likelihood of malignancy.

## 4 MRI Signatures of Parotid Tumours

Each of the histopathological types of parotid tumours was compared with morphology and signal intensity–related variables, and a significant difference (*p*‐value < 0.05) was seen with size, margins, capsule, fat component, any T2 component hypointense to gland, cyst component, diffusion restriction, contrast enhancement, predominant T2 hyperintensity in the solid component and extraglandular extension. Histopathological types of tumours which were ≥ 3 in number were analysed to see if MRI signatures could be assigned to them based on the predominant or unique morphology and signal intensity–related findings. The fat component was completely absent in adenoid cystic carcinoma (AdCC), intermediate and high‐grade MEC, poorly differentiated carcinoma and non‐Hodgkin lymphoma (NHL). Location purely in the deep lobe was seen for PA [2/13 (15.4%)], carcinoma ex‐PA [2/9 (22.2%)] and epithelial myoepithelial carcinoma [1/3 (33.3%)]. Location in the parotid tail was seen in WT [7/15 (46.6%)], carcinoma ex‐PA [1/9 (11.1%)], PA [1/13 (7.7%)] and Grade II AdCC [1/4 (25%)].

### 4.1 WT

**FIGURE 1:**
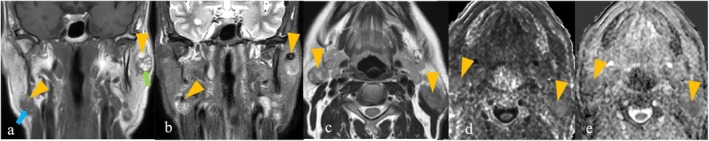
(a–e) 66‐year‐old male patient with bilateral Warthin tumours. (a) Coronal T1WI shows well‐defined oval shaped tumours in superficial lobe (parotid tail) of right parotid gland (blue arrow) and superficial lobe of left parotid gland (green arrow), showing T1 hyperintensity (arrowheads) which are supressed on STIR sequence (arrowheads in b), suggestive of fat content. (c) Axial T2WI shows hypointense components within these tumours (arrowheads) which are hypointense to parotid gland but not isointense to muscle. These tumours show patchy diffusion restriction seen as hyperintense on DWI (arrowheads in d) with signal drop on ADC (arrowheads in e).

All were male. Three patients had bilateral tumours. As shown in Table S4 (in supplementary file 4), encapsulated, oval‐shaped < 4‐cm‐sized tumours located in parotid tail with presence of TI hyperintensity and fat, showing diffusion restriction, presence of T2 signal hypointense to gland but not to muscle and absence of extraglandular extension, are typical for WT (Figure 1). These tumours may show hypoenhancement or rim enhancement.

### 4.2 PA

**FIGURE 2:**
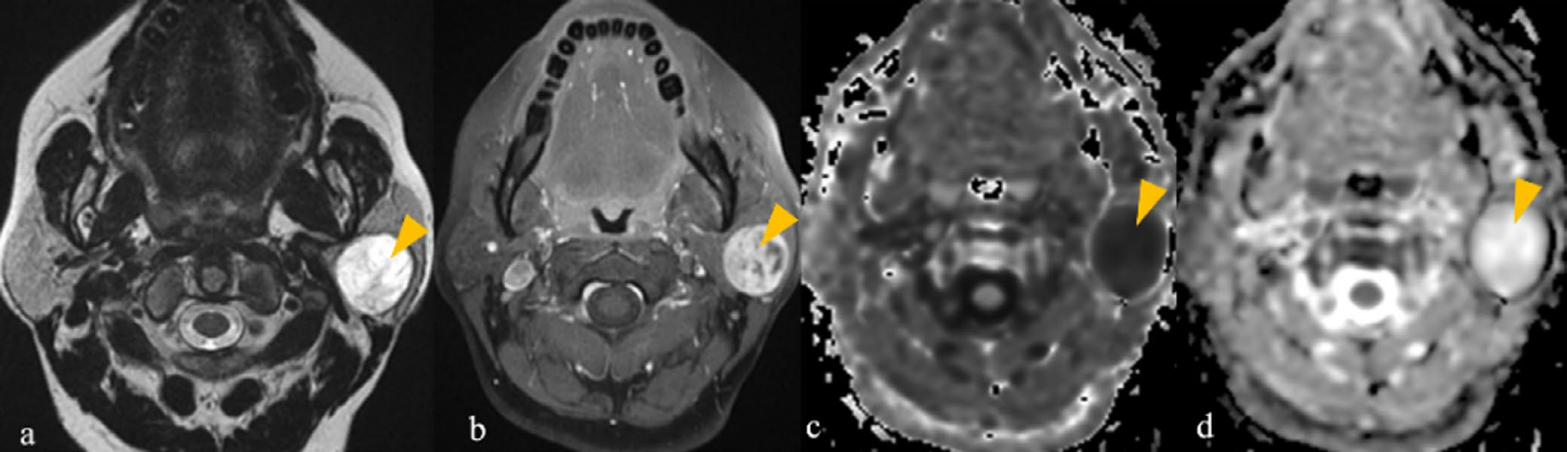
(a–d) 48‐year‐old female patient with Pleomorphic adenoma. (a) Axial T2WI shows a well‐defined encapsulated round‐shaped tumour in the superficial lobe of left parotid gland showing predominant hyperintense signal intensity (arrowhead). (b) Postcontrast T1WI shows heterogeneous enhancement (arrowhead). DWI (c) and ADC (d) show absence of diffusion restriction (arrowheads).

As shown in Table S5 (in supplementary file 4), encapsulated round‐shaped tumour with well‐defined lobulated margins showing predominant T2 hyperintensity in the solid component, heterogeneous enhancement or rim enhancement, without any fat or T2 hypointense signal or diffusion restriction, is typical for PA (Figure 2). Extraglandular extension, but not infiltration, may be seen in large tumours. PA can be located purely in the deep lobe as well.

### 4.3 MEC

**FIGURE 3:**
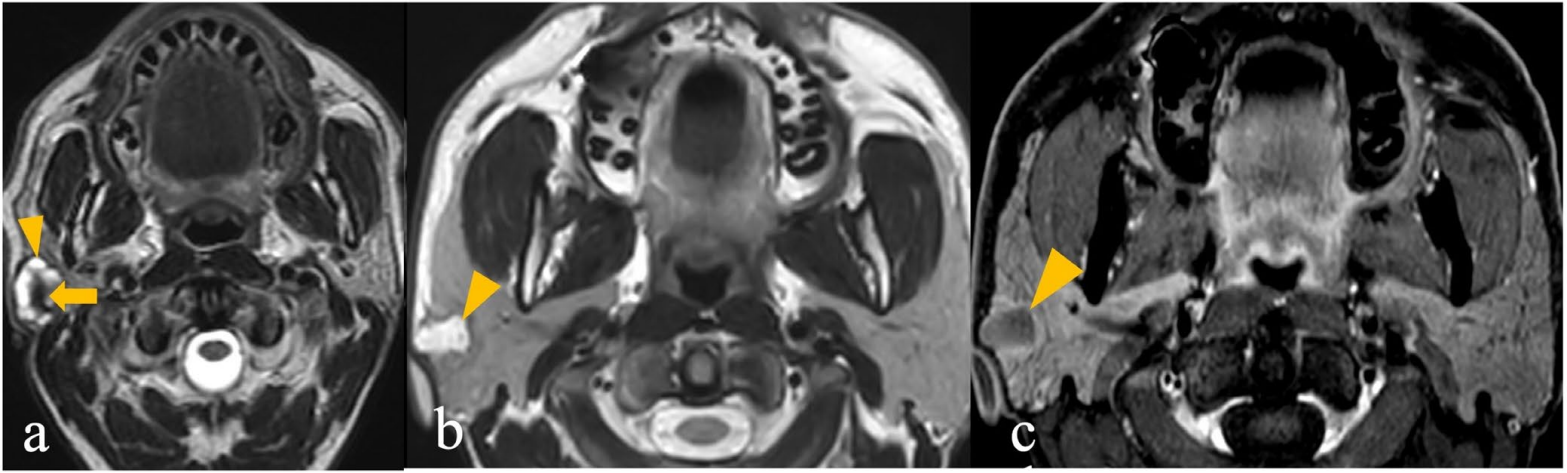
(a–c) 60‐year‐old male patient (a) and 39‐year‐old male patient (b and c) with two different patterns of low‐grade Mucoepidermoid carcinoma. (a) Axial T2WI shows an encapsulated tumour with well‐defined lobulated margins in superficial lobe of right parotid gland, showing predominant T2 hyperintensity in the solid component (arrowhead), along with presence of T2 hypointensity isointense to muscle (arrow). (b and c) Another patient shows encapsulated irregular shaped tumour in the superficial lobe of right parotid gland on axial T2WI (b) with presence of cyst (arrowhead), and rim enhancement on post contrast axial T1WI (c) (arrowhead).

As shown in Table S6 (in supplementary file 4), irregular‐shaped, encapsulated tumour with well‐defined lobulated margins, predominant T2 hyperintensity in the solid component, presence of T2 hypointense component isointense to muscle (Pattern 1) or presence of cyst with rim enhancement (pattern 2), are typical for low‐grade MEC (Figure 3). Fat is absent in the majority (11 out of 12) of the low‐grade MEC.

Tumour with/without infiltrative margins, location both in superficial and deep lobes, containing cyst and T2 signal intensity hypointense to gland but not isointense to muscle, lacking predominant T2 hyperintensity in the solid component, showing absence of fat and lacking rim enhancement, showing homogeneous or heterogeneous contrast enhancement, are typical for intermediate/high‐grade MEC (Figure S1) (in supplementary file 2).

T1 hyperintensity can be present in any grade of MEC.

### 4.4 Salivary Duct Carcinoma (SDC)

All patients were male. As shown in Table S7 (in supplementary file 4), an unencapsulated tumour with irregular shape, infiltrative margins, presence of T1 hyperintensity, presence of T2 signal hypointense to gland, presence of cyst, absence of predominant T2 hyperintensity in the solid component, showing heterogeneous contrast enhancement and extraglandular extension, is typical for SDC (Figure S2) (in supplementary file 2). Diffusion restriction was absent in 2/11 patients (18.2%).

### 4.5 Carcinoma ex Pleomorphic Adenoma (Ca ex PA)

As shown in Table S8 (in supplementary file 4), > 2‐cm‐sized irregular‐shaped tumour with either well‐defined lobulated or part‐infiltrative and part‐lobulated margins, with presence of fat, some T2 signal hypointense to gland as well as isointense to muscle, showing diffusion restriction, and heterogeneous contrast enhancement, is typical for Ca ex PA (Figure S3) (in supplementary file 2).

### 4.6 AdCC

All the three grades of AdCC (*n* = 6) were > 2 cm in size, showed strong enhancement and none of them had a fat component. The majority of the AdCC showed infiltrative margins (5/6 [83.33%]), extraglandular extension (4/6 [66.66%]) and absence of T2 hypointense component (4/6 [66.66%]). Three out of six (50%) showed diffusion restriction, and 2/6 (33.33%) showed the presence of a cyst. Figure S4 (in supplementary file 2) shows a typical case of AdCC.

### 4.7 Mammary Analogue Secretory Carcinoma (MASC)

As shown in Table S9 (in supplementary file 4), > 2‐cm‐sized hypoenhancing tumour without any infiltrative or ill‐defined margins, showing cyst with papillary projections showing diffusion restriction, presence of T1 hyperintensity with/without fat and lacking rim enhancement, is typical for MASC (Figure S5) [in supplementary file 2].

### 4.8 NHL

None of the four tumours were < 2 cm in size, none of them had fat or capsule, none of them showed predominant T2 hyperintensity in the solid component or hypointensity isointense to muscle. All the four NHL showed homogeneous diffusion restriction. Fifty per cent of the NHL showed hypoenhancement and 50% showed heterogeneous enhancement.

**FIGURE 4:**
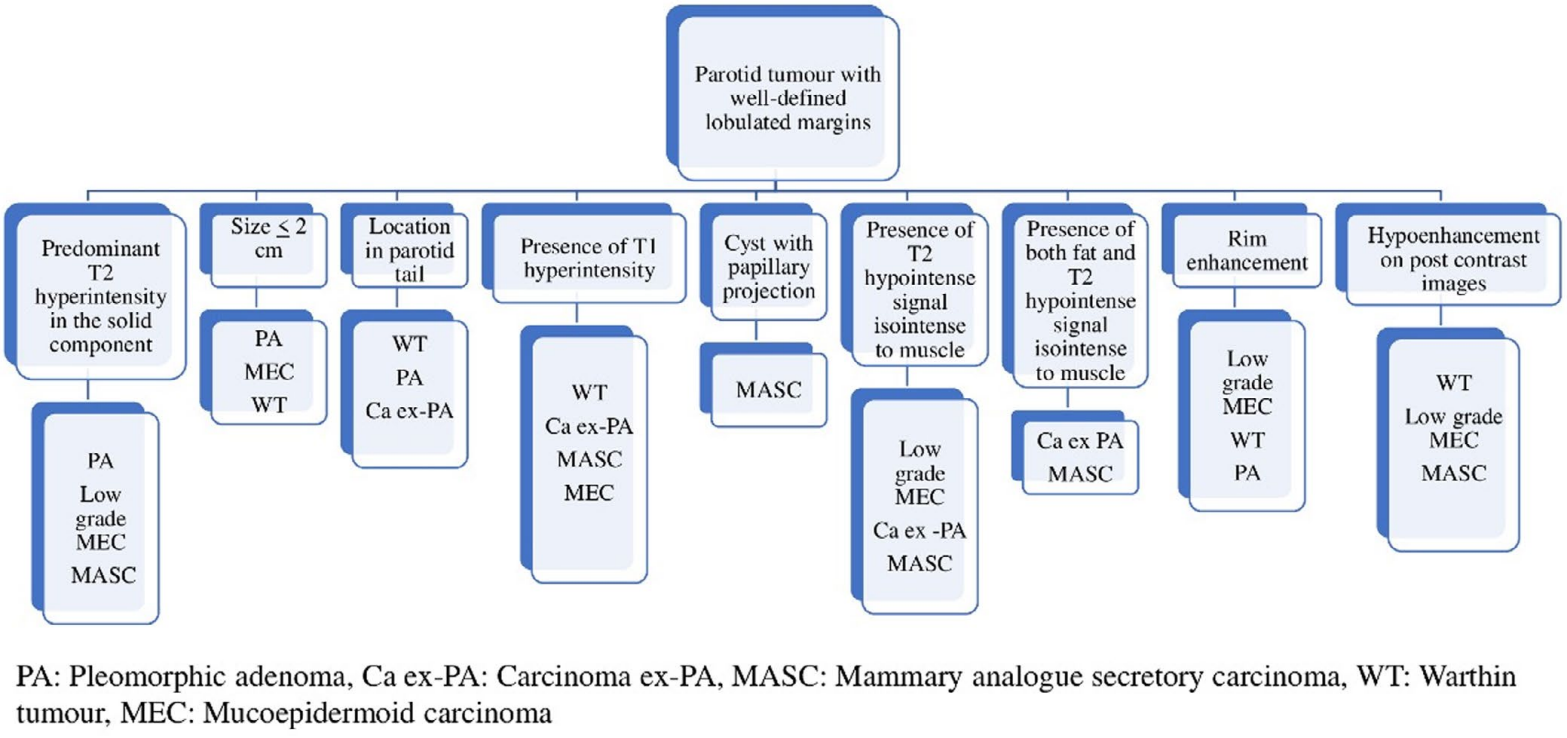
Algorithm for reaching to a histopathological diagnosis of a parotid tumour with well‐defined lobulated margins, based on the MRI signatures.

**FIGURE 5:**
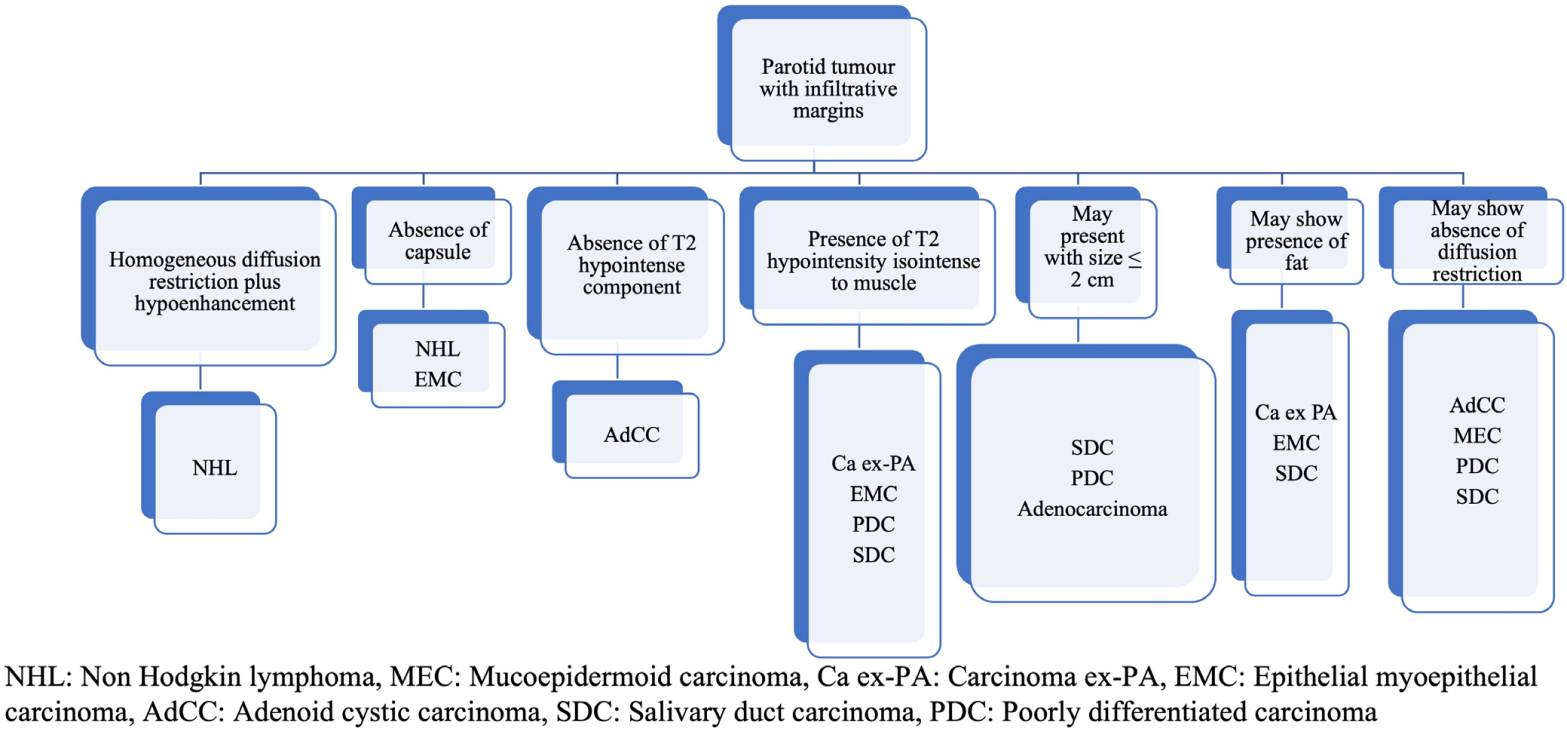
Algorithm for reaching a histopathological diagnosis of a parotid tumour with infiltrative margins, based on the MRI signatures.

Results show that well‐defined lobulated margins can be seen in both benign as well as malignant parotid tumours, and so, based on the MRI signatures, we have prepared an algorithm (Figure 4) to be followed for reaching a histopathological diagnosis of a parotid tumour with well‐defined lobulated margins. Parotid tumours with infiltrative margins are malignant and the algorithm in Figure 5 shows the likely malignant histopathology based on the MRI signatures.

We have prepared an MRI reporting template (supplementary file 1) for the baseline evaluation of parotid tumours based on the MRI signatures of benign and malignant parotid tumours.

## 5 Validation

Sensitivity, specificity, accuracy, PPV and NPV for benign and malignant nature of parotid tumours predicted by Radiologist 3 using the developed MRI signatures, flowcharts and MRI reporting template were 92.31%, 100%, 94.23%, 100% and 81.25%, respectively, with a highly significant *p*‐value of < 1e‐04 (Table S10) [in supplementary file 4]. High statistical performance was also observed for WT, PA, low grade and intermediate/high‐grade MEC, MASC, Ca ex‐PA, SDC, AdCC, NHL (Tables S11–S19) [in supplementary file 4], of which WT, PA, low‐grade MEC, MASC and NHL showed a highly significant *p*‐value of < 1e‐04.

## 6 Discussion

To the best of our knowledge, there has been no study so far assigning MRI signatures to various benign and malignant parotid histopathologies, including grades of MEC, using so many morphology and signal intensity–related variables. MRI variables such as presence or absence of fat component, subdivision of tumour T2 SI hypointense to gland into two variables; with or without isointense to muscle, patterns of diffusion restriction into homogeneous/patchy/part of the lesion, and postcontrast rim enhancement, have been evaluated for the first time in our study. T1 hyperintensity has been evaluated for only WT in the existing literature, whereas we have analysed this variable in all the benign and malignant tumours in our study.

PA was the most common benign parotid tumour and MEC was the most common malignant parotid tumour in our study, consistent with the findings in the existing literature [3].

Ali et al. had the largest HPR proven malignant parotid tumour sample size of 79; however, they did not assign MRI signatures to various malignant parotid tumours [10].

Irregular shape and extraglandular infiltrative pattern were seen only in malignant parotid tumours, similar to the findings by Tartaglione et al. and Yerli et al. [11, 12]. In our study, the presence of a capsule was significantly associated with a benign histopathology, similar to the findings in literature [11]. Our study showed lobulated margins and T2 hyperintensity in PA, and T1 hyperintensity and postcontrast hypoenhancement in WT, which was in agreement with the findings of previous studies [11, 13, 14]. Significant correlation of T2 hypointensity with malignant parotid tumour in our study was similar to studies by Elmokadem et al. and Stoia et al. [13, 15]. Similar to the findings by Elmokadem et al., the majority of our malignant parotid tumours were located both in superficial and deep lobes [15].

We found T2 hyperintense signal as well as ill‐defined margins in low‐grade MEC in our study, which was consistent with the findings by Kashiwagi et al. [16]. MASC is the only tumour that showed a cyst with papillary projections in our study, which was consistent with the findings in the study by Kashiwagi et al. [17]. In our study, all the grades of AdCC showed strong contrast enhancement, consistent with the findings in the literature [2]. The majority of the Ca ex‐PA in our study showed a T2 hypointense component and diffusion restriction, consistent with the findings in the literature [2, 18].

Our study showed infiltrative margins, a cystic component and T2 hypointensity in SDC, consistent with the study by Weon et al. [19].

Contrary to the study by Freling et al. [20], we found a significant difference between MRI features of benign and malignant tumours and also found typical MRI signatures of various parotid gland tumours including grades of MEC. However, their sample size was only 30 for malignant tumours; they did not study contrast enhancement and other variables included in our study.

Small sample size was a limitation in our study. Radiological findings could not be correlated with intraoperative findings due to haphazard surgical notes lacking pertinent information. The template for systematically recording intraoperative findings for parotid tumour surgery is attached in the supplementary file 3. Studies have shown the role of advanced MRI techniques, such as dynamic contrast‐enhanced MRI, quantitative ADC values and intravoxel incoherent motion (IVIM), for parotid tumour differentiation; however, we do not routinely perform these sequences in our institute for parotid imaging; hence, unfortunately, we could not incorporate these techniques into our algorithm [21, 22, 23, 24].

## 7 Conclusion

Significant *p*‐value and high statistical performance were observed for benign and malignant nature of parotid tumours and parotid tumour histopathologies using the developed MRI signatures. Infiltrative margins/extraglandular extension with invasion into surrounding structures/metastatic cervical nodes/T2 hypointense component that is isointense to muscle/cyst with papillary projections suggest malignant aetiology. T2 signal intensity and enhancement patterns can help identify low‐grade MEC, impacting management decisions regarding END.

## Author Contributions

Nivedita Chakrabarty: Conceptualisation, Data Curation, Methodology, Writing‐original draft, Writing‐review and editing. Prathamesh Pai: Conceptualisation, Writing‐review and editing. Arpita Sahu: Writing‐review and editing. Oindrila Roy Chowdhury: Formal analysis. Pashmina Kandalgaonkar: Data Curation. Tapish Dadlani: Validation. Munita Menon: Writing‐review and editing. Suman Kumar Ankathi: Conceptualisation. All authors read and approved the submitted version.

## Ethics Statement

This study has been approved by the Institutional Ethical Committee of Tata Memorial Centre, Homi Bhabha National Institute (HBNI), Parel, Mumbai, Maharashtra, 400,012, India, with the project ID 900954.

## Consent

Consent to participate was waived off by the Institutional Ethical Committee as this was a retrospective study and patient information was not revealed.

## Conflicts of Interest

The authors declare no conflicts of interest.

## Supporting information


**Data S1** Supporting Information.

**Data S2** Supporting Information.

**Data S3** Supporting Information.

**Data S4** Supporting Information.

## Data Availability

Data sharing is not applicable to this article as no new data were created or analyzed in this study. Original contributions in the study have been included in the article. Further enquiries can be directed to the corresponding author.
