# Supplementary material for: MRI Signatures of Parotid Tumours Impacting Management Decisions: A Retrospective Study With Radiology and Pathology Correlation
Source: J Med Imaging Radiat Oncol. 2025 May 19;69(4):452–61. doi: 10.1111/1754-9485.13865 (PMC12175207; doi:10.1111/1754-9485.13865)
Supplement: Supplementary file 1 — Data S1 Supporting Information. [file ARA-69-452-s001.docx]

**MRI reporting template for baseline evaluation of parotid tumour**

Clinical history:

Age: Sex:

Clinical examination findings:

MRI sequences:

**Findings:**

Laterality: Right/Left/Bilateral

Location of tumour within parotid gland: Superficial lobe/Deep lobe/Both/Parotid tail

Size of tumour: < 2 cm/ > 2 to < 4 cm/ > 4 cm

Capsule: Present/Absent

Margins: Well-defined smooth/Well-defined lobulated/Infiltrative

Shape: Oval/Round/Irregular

T1 hyperintense component: Present/Absent

Fat component (T1 hyperintensity suppressed on STIR): Present/Absent

Predominant T2 hyperintensity within the solid component of parotid tumour: Present/Absent

Any T2 signal intensity hypointense to parotid gland: Present/Absent

If yes, T2 hypointense signal isointense to muscle: Present/Absent

Cyst with papillary projections: Present/Absent

Pattern of diffusion restriction: Absent/Patchy/Homogeneous

Pattern of contrast enhancement: Hypoenhancing/Rim enhancing/ Heterogeneous/ Homogeneous

Extraglandular extension: Present/Absent Structures infiltrated (if present):

Perineural spread: Present/Absent Nerve involved (if present):

Intracranial extension of tumour/Perineural spread: Present/Absent

Bone involved: Yes/ No/Indeterminate* Name of bone/bones involved:

Metastatic neck nodes: Present/Absent/Indeterminate^$^

Level of nodes, if involved:

Size of nodes (if involved):

Extranodal extension (ENE): Present/Absent

Other findings:

Impression: Benign/Indeterminate/Malignant

Likely histopathology (if possible to comment):

Radiological stage (if malignant):

* CT required for further evaluation

$ USG guided FNA required
