# Supplementary material for: MRI Signatures of Parotid Tumours Impacting Management Decisions: A Retrospective Study With Radiology and Pathology Correlation
Source: J Med Imaging Radiat Oncol. 2025 May 19;69(4):452–61. doi: 10.1111/1754-9485.13865 (PMC12175207; doi:10.1111/1754-9485.13865)
Supplement: Supplementary file 2 — Data S2 Supporting Information. [file ARA-69-452-s003.docx]

**Supplementary figures**

*
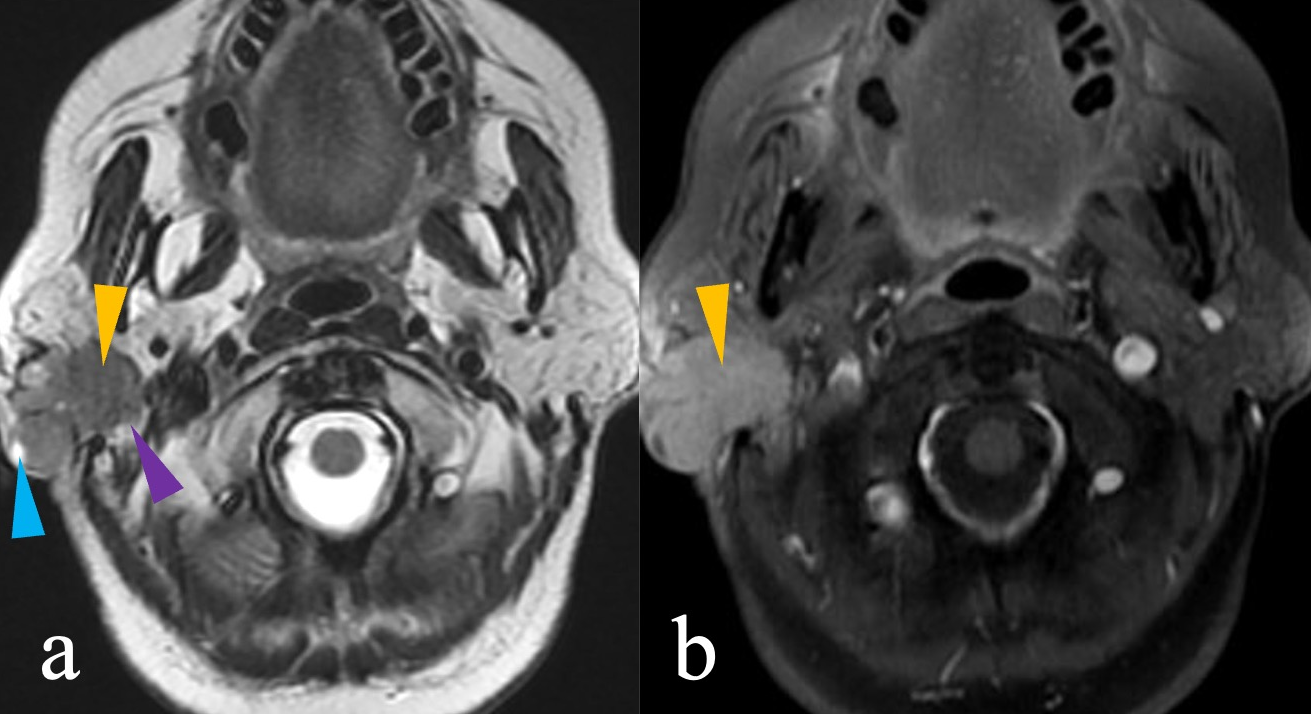
*

**Supplementary figure 1 (a and b)** 60 years old female patient with high grade Mucoepidermoid carcinoma (MEC). a) Axial T2WI shows a well defined irregular shaped lesion involving both superficial and deep lobes of right parotid gland with extraglandular extension (purple arrowhead) showing T2 signal intensity hypointense to gland (yellow arrowhead) but not isointense to muscle signal, and few T2 hyperintense cystic component (blue arrowhead). b) The solid component shows homogeneous enhancement (arrowhead).


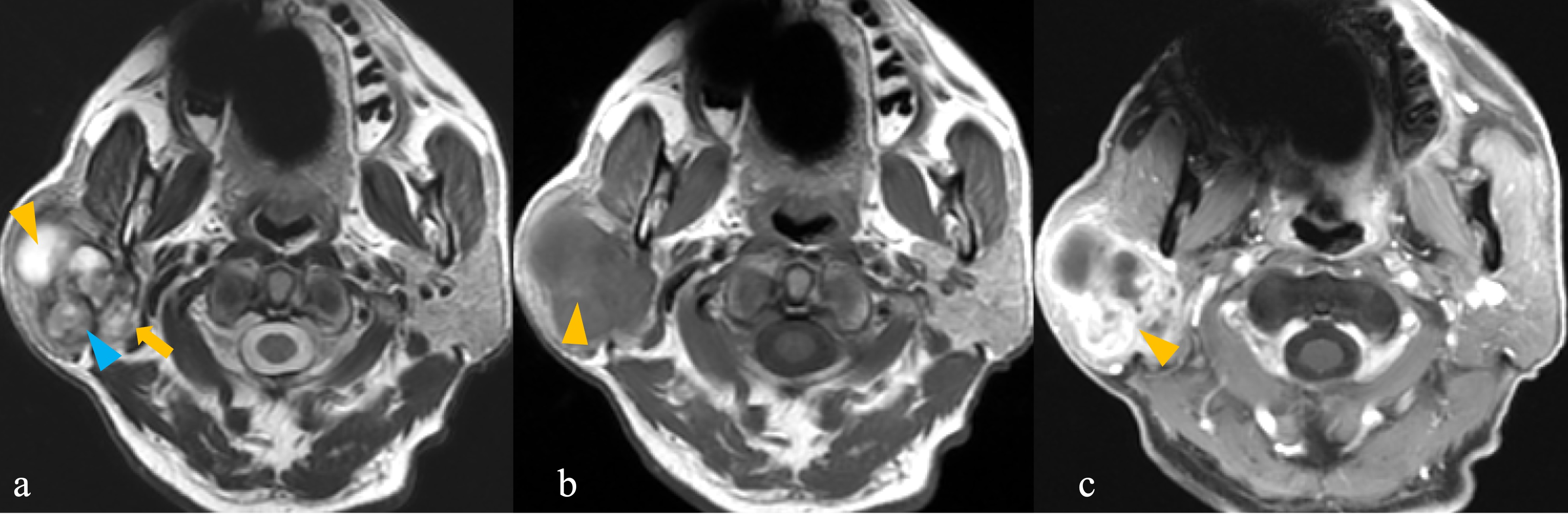


**Supplementary figure 2 (a-c)** 62 years old male with salivary duct carcinoma. a) Axial T2WI shows an irregular shaped tumour in right parotid gland occupying both superficial and deep lobe, showing T2 hyperintense cyst (yellow arrowhead) and T2 signal hypointense to gland (blue arrowhead). In addition, infiltrative margins with extraglandular extension is seen (arrow). b) Axial T1WI shows hyperintense components within (arrowhead). Axial post contrast T1WI shows heterogeneous enhancement (arrowhead)

*
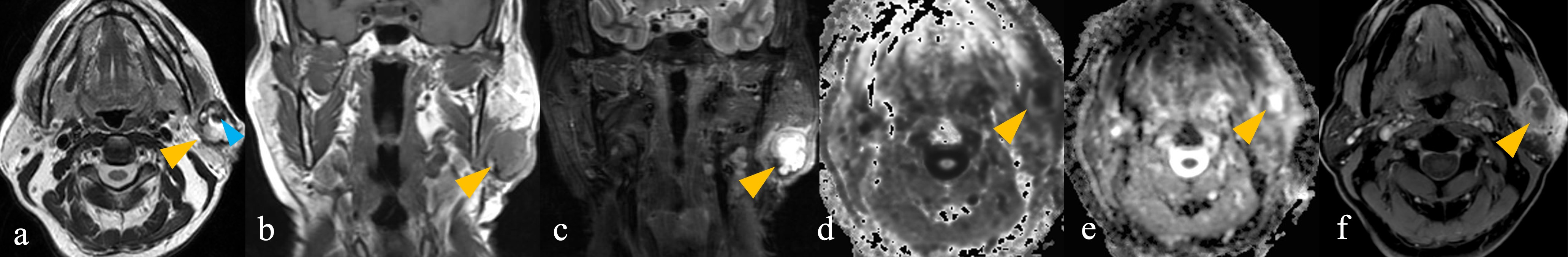
*

**Supplementary figure 3 (a-f)** 61 years male patient with Carcinoma ex-Pleomorphic adenoma. a) Axial T2WI shows an irregular shaped tumour with part-lobulated part-infiltrative margins (yellow arrowhead) in the superficial lobe of left parotid gland showing T2 hypointense component isointense to muscle (blue arrowhead). Axial T1WI (b) shows hyperintense component within the tumour (arrowhead) which shows suppression on STIR (arrowhead in c) suggestive of fat. DWI (d) and ADC (e) show patchy diffusion restriction (arrowheads). f) Post contrast T1WI shows heterogeneous enhancement of the tumour (arrowhead)


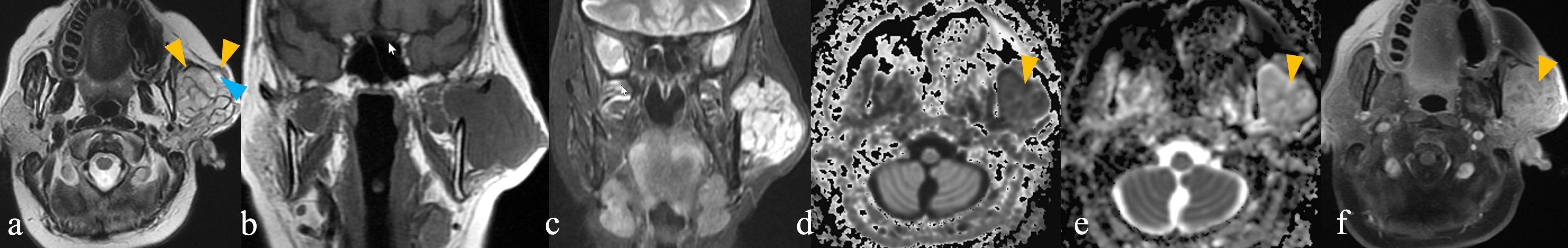


**Supplementary figure 4 (a-f)** 61 years old female patient with Adenoid cystic carcinoma. a) Axial T2WI shows a tumour with irregular shape and infiltrative margins in the superficial lobe of left parotid gland showing extraglandular extension (yellow arrowheads). The tumour shows signal intensity isointense to parotid gland without any hypointense component. Small T2 hyperintense cystic component is seen (blue arrowhead). Axial T1WI (b) and STIR (c) reveal absence of fat. DWI (d) and ADC (c) show patchy diffusion restriction (arrowheads). f) Post contrast T1WI shows intense heterogeneous contrast enhancement (arrowhead)

***
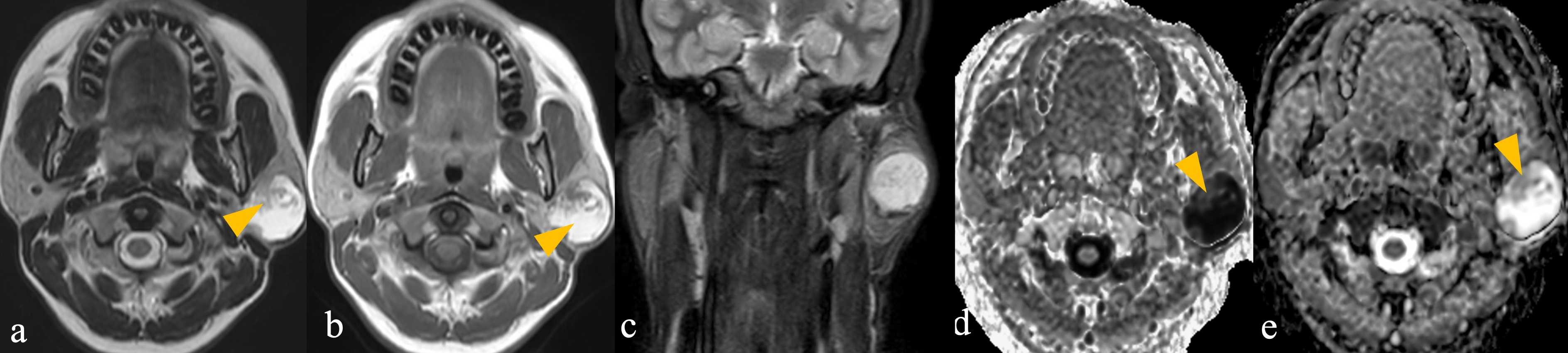
***

**Supplementary figure 5 (a-e)** 41 years old male patient with Mammary analogue of secretory carcinoma. a) Axial T2WI shows a well defined tumour in the superficial lobe of left parotid gland containing hyperintense cyst with papillary projections (arrowhead). b) Axial T1WI shows hyperintense content (arrowhead) which does not suppress on STIR (c), suggesting absence of fat. DWI (d) and ADC (e) show diffusion restriction of the papillary projections (arrowheads)
