# Supplementary material for: MRI Signatures of Parotid Tumours Impacting Management Decisions: A Retrospective Study With Radiology and Pathology Correlation
Source: J Med Imaging Radiat Oncol. 2025 May 19;69(4):452–61. doi: 10.1111/1754-9485.13865 (PMC12175207; doi:10.1111/1754-9485.13865)
Supplement: Supplementary file 3 — Data S3 Supporting Information. [file ARA-69-452-s004.docx]

**Template for recording intraoperative findings of parotid tumour surgery**

Patient’s name: Gender:

Patient ID No.:

Histopathological Diagnosis:

Type of surgery:

Type of neck dissection (if performed):

Date of surgery:

***Intraoperative findings:***

Location of tumour within the parotid gland: superficial lobe/deep lobe/both

***Intraoperative relation of tumour to the following key structures:***

1. Facial nerve: free/ encased but can be removed/involved

2. Stylomandibular foramen: free/involved

3. Retromandibular vein: free/displaced/involved

4. External carotid artery branches: free/displaced/involved

5. Styloid process: free/involved

6. Extraglandular spread: Present/Absent

7. Masseter muscle: free/involved

8. Mandible: free/involved

9. Skin: free/involved

10. Other findings:

***Procedure:***
