# Supplementary material for: MRI Signatures of Parotid Tumours Impacting Management Decisions: A Retrospective Study With Radiology and Pathology Correlation
Source: J Med Imaging Radiat Oncol. 2025 May 19;69(4):452–61. doi: 10.1111/1754-9485.13865 (PMC12175207; doi:10.1111/1754-9485.13865)
Supplement: Supplementary file 4 — Data S4 Supporting Information. [file ARA-69-452-s002.docx]

**Supplementary tables**

**Supplementary table 1: Mean age and sex distribution**

| Variable | Level | Malignant tumours  (n=72) | Benign  tumours  (n=26) | Total (n=98) |
| --- | --- | --- | --- | --- |
| Age (in years) | mean (SD) | 49.1 (16) | 53.3 (17.6) | 50.2 (16.5) |
| Sex |  |  |  |  |
| Female | frequency (percentage) | 22 (30.6) | 12 (46.2) | 34 (34.7) |
| Male | frequency (percentage) | 50 (69.4) | 14 (53.8) | 64 (65.3) |

**Supplementary table 2: Benign parotid gland tumours with the number of lesions in each of these categories**

| **Histopathological type of benign parotid tumour** | **Number of lesions**  **n=32** |
| --- | --- |
| Warthin tumour | 15 (9 patients) |
| Pleomorphic adenoma | 13 |
| Basal cell adenoma | 1 |
| Lipoblastoma | 1 |
| Oncocytoma | 1 |
| Paraganglioma | 1 |

**Supplementary table 3: Malignant parotid gland tumours with the number of lesions in each of these categories**

| **Histopathological type of malignant parotid tumour** | **Number of lesions**  **(n=72)** |
| --- | --- |
| *Mucoepidermoid carcinoma*  Mucoepidermoid carcinoma low grade (n=12)  Mucoepidermoid intermediate grade (n=5)  Mucoepidermoid carcinoma high grade (n=2) | 19 |
| Carcinoma ex-pleomorphic adenoma | 9 |
| Salivary duct carcinoma | 11 |
| Mammary analogue secretory carcinoma | 6 |
| *Adenoid cystic carcinoma*  Adenoid cystic carcinoma, grade I (n=1)  Adenoid cystic carcinoma, grade II (n=4)  Adenoid cystic carcinoma, grade III (n=1) | 6 |
| Acinic cell carcinoma | 1 |
| Non-Hodgkin lymphoma | 4 |
| Neuroendocrine tumor | 1 |
| *Adenocarcinoma*  Polymorphous low grade adenocarcinoma (n=1)  Adenocarcinoma, not otherwise specified (n=1) | 2 |
| Synovial sarcoma | 1 |
| Epithelial myoepithelial carcinoma | 3 |
| Ewings sarcoma | 2 |
| Poorly differentiated carcinoma | 4 |
| Keratinizing squamous cell carcinoma | 1 |
| Lymphoepithelial carcinoma | 2 |

**Supplementary table 4: MRI signatures of Warthin tumour**

| **MRI findings** | **Number of lesions (n)=15** |
| --- | --- |
| Oval shape | 8 (53.33%) |
| Size> 4cm | 0 |
| Location in parotid tail | 7 (46.6%) |
| Presence of capsule | 14 (93.33%) |
| Well-defined smooth margins | 6 (40%) |
| Well-defined lobulated margins | 5 (33.3%) |
| T1 hyperintensity | 13 (86.66%) |
| Presence of fat | 9 (60%) |
| Presence of T2 signal hypointense to gland | 13 (86.66%) |
| Any T2 signal hypointense to gland and isointense to muscle | 0 |
| Presence of predominant T2 hyperintensity in the solid component | 3 (20%) |
| Patchy diffusion restriction | 9 (60%) |
| Homogeneous diffusion restriction | 5 (33.33%) |
| Hypoenhancement | 7 (46.7%) |
| Extraglandular extension | 0 |

**Supplementary table 5: MRI signatures of Pleomorphic adenoma**

| **MRI findings** | **n=13** |
| --- | --- |
| Round shape | 7 (53.8%) |
| Well-defined lobulated margins | 11 (84.6%) |
| Presence of capsule | 13 (100%) |
| Presence of cyst | 3 (23.07%) |
| Predominant T2 hyperintensity in the solid component | 11 (84.6%) |
| Presence of fat component | 1 (7.69%) |
| Presence of T2 signal hypointense to gland | 3 (23.07%) |
| Presence of T2 hypointensity isointense to muscle | 0 |
| Presence of patchy diffusion restriction | 5 (38.46%) |
| Presence of homogeneous diffusion restriction | 0 |
| Heterogeneous contrast enhancement | 10 (76.9%) |
| Presence of rim enhancement | 2 (15.4%) |
| Extraglandular extension but not infiltration | 2 (15.4%) |

**Supplementary table 6: MRI signatures of grades of Mucoepidermoid carcinoma**

| **MRI findings** | **Low grade MEC**  **(n=12)** | **Intermediate grade MEC**  **(n=5)** | **High grade MEC**  **(n=2)** |
| --- | --- | --- | --- |
| Location in superficial and deep lobe | 4 (33.3%) | 3 (60%) | 2 (100 %) |
| Irregular shape | 8 (66.7%) | 2 (40%) | 2 (100 %) |
| Well defined lobulated margins | 7 (58.3%) | 3 (60%) | 0 |
| Infiltrative or part lobulated and part infiltrative | 2 (16.7%) | 1 (20%) | 2 (100%) |
| Ill defined margins | 2 (16.7%) | 1 (20.0%) | 0 |
| Presence of complete or interrupted capsule | 11 (91.7%) | 4 (80%) | 2 (100%) |
| Presence of cyst | 6 (50%) | 3 (60%) | 2 (100%) |
| Presence of T1 hyperintensity | 6 (50%) | 2 (40%) | 1 (50%) |
| Presence of fat | 1 (8.33%) | 0 | 0 |
| Predominant T2 hyperintensity in the solid component | 4 (33.3%) | 0 | 0 |
| Presence of T2 signal hypointense to gland | 9 (75%) | 5 (100%) | 1 (50%) |
| Presence of T2 hypointensity isointense to muscle | 3 (25%) | 0 | 0 |
| Patchy or homogeneous diffusion restriction | 7 (58.3%) | 4 (80%) | 1 (50%) |
| Absence of diffusion restriction | 5 (41.7%) | 1 (20%) | 1 (50%) |
| Homogeneous contrast enhancement | 6 (50%) | 3 (60%) | 1 (50%) |
| Rim enhancement | 4 (33.3%) | 0 | 0 |
| Hypoenhancement | 1 (8.3%) | 0 | 0 |
| Heterogeneous contrast enhancement | 1 (8.3%) | 2 (40%) | 1 (50%) |
| Extraglandular extension | 4 (33.3%) | 2 (40%) | 1 (50%) |

**Supplementary table 7: MRI signatures of grades of Salivary duct carcinoma**

| **MRI findings** | **n=11** |
| --- | --- |
| Irregular shape | 10 (90.9%) |
| Infiltrative margins | 8 (72.7%) |
| Presence of capsule | 4(36.36%) |
| Presence of cyst | 5 (45.5%) |
| T1 hyperintensity | 6 (54.5%) |
| Presence of fat | 2 (18.2%) |
| Any T2 signal hypointense to gland | 10 (90.9%) |
| Any T2 signal hypointense to gland and isointense to muscle | 1 (9.1%) |
| Heterogeneous contrast enhancement | 10 (90.9%) |
| Predominant T2 hyperintensity in the solid component | 1 (9.09%) |
| Extraglandular extension | 8 (72.7%) |

**Supplementary table 8: MRI signatures of Carcinoma ex pleomorphic adenoma**

| **MRI findings** | **n=9** |
| --- | --- |
| Size < 2 cm | 0 |
| Irregular shape | 6 (66.7%) |
| Well-defined lobulated margins | 3 (33.3%) |
| Infiltrative | 3 (33.3%) |
| Part infiltrative, part lobulated | 2 (22.2%) |
| Presence of capsule | 5 (55.55%) |
| Presence of T1 hyperintensity and fat | 3 (33.3%) |
| Any T2 signal hypointense to gland | 8 (88.9%) |
| Any T2 signal hypointense to gland and isointense to muscle | 5 (55.6%) |
| Predominant T2 hyperintensity in the solid component | 2 (22.22%) |
| Diffusion restriction which is patchy or in part of the tumour | 8 (88.9%) |
| Heterogeneous contrast enhancement | 9 (100%) |
| Extraglandular extension | 5 (55.6%) |

**Supplementary table 9: MRI signatures of Mammary analogue secretory carcinoma**

| **MRI findings** | **n=6** |
| --- | --- |
| Size < 2 cm | 0 |
| Presence of cyst with papillary projections | 4 (66.7%) |
| Infiltrative margins | 0 |
| Ill-defined margins | 0 |
| Presence of T1 signal hyperintensity | 5 (83.3%) |
| Presence of fat | 1 (16.7%) |
| Presence of T2 signal hypointense to gland | 2 (33.3%) |
| Presence of T2 signal isointense to muscle | 1 (16.7%) |
| Predominant T2 hyperintensity in the solid component | 2 (33.3%) |
| Presence of diffusion restriction | 6 (100%) |
| Hypoenhancement | 4 (66.7%) |
| Rim enhancement | 0 |
| Extraglandular extension | 4 (66.7%) |

**Supplementary table 10: Prediction of benign versus malignant nature of parotid tumour by Radiologist 3 using MRI signatures**

| Variable | Level | Malignant (n=78) | Benign (n=26) | Total (n=104) | p-value |
| --- | --- | --- | --- | --- | --- |
| Actual histopathology | Malignant | 72 (92.3) | 0 (0.0) | 72 (69.2) |  |
|  | Benign | 6 (7.7) | 26 (100.0) | 32 (30.8) | < 1e-04 |

| Statistics | Value | 95% CI |
| --- | --- | --- |
| Sensitivity | 92.31% | 84.01% to 97.12% |
| Specificity | 100.00% | 86.77% to 100.00% |
| Positive Predictive Value | 100.00% | 95.01% to 100.00% |
| Negative Predictive Value | 81.25% | 66.76% to 90.34% |
| Accuracy | 94.23% | 87.87% to 97.85% |

**Supplementary table 11: Prediction of Warthin tumour by Radiologist 3 using MRI signatures**

| Variable | Level | Others (n=90) | Warthin Tumour (n=14) | Total (n=104) | p-value |
| --- | --- | --- | --- | --- | --- |
| Actual histopathology | Others | 89 (98.9) | 0 (0.0) | 89 (85.6) |  |
|  | Warthin Tumour | 1 (1.1) | 14 (100.0) | 15 (14.4) | < 1e-04 |

| **Statistic** | **Value** | **95% CI** |
| --- | --- | --- |
| Sensitivity | 100.00% | 76.84% to 100.00% |
| Specificity | 98.89% | 93.96% to 99.97% |
| Positive Predictive Value | 93.33% | 66.60% to 98.99% |
| Negative Predictive Value | 100.00% | 95.94% to 100.00% |
| Accuracy | 99.04% | 94.76% to 99.98% |

**Supplementary table 12: Prediction of Pleomorphic adenoma by Radiologist 3 using MRI signatures**

| Variable | Level | Others (n=93) | Pleomorphic adenoma (n=11) | Total (n=104) | p-value |
| --- | --- | --- | --- | --- | --- |
| Actual histopathology | Others | 89 (95.7) | 2 (18.2) | 91 (87.5) |  |
|  | Pleomorphic adenoma | 4 (4.3) | 9 (81.8) | 13 (12.5) | < 1e-04 |

| **Statistics** | **Value** | **95% CI** |
| --- | --- | --- |
| Sensitivity | 81.82% | 48.22% to 97.72% |
| Specificity | 95.70% | 89.35% to 98.82% |
| Positive Predictive Value | 69.23% | 45.33% to 85.93% |
| Negative Predictive Value | 97.80% | 92.70% to 99.36% |
| Accuracy | 94.23% | 87.87% to 97.85% |

**Supplementary table 13: Prediction of Low grade Mucoepidermoid carcinoma by Radiologist 3 using MRI signatures**

| Variable | Level | Others (n=89) | Mucoepidermoid carcinoma, low grade (n=15) | Total (n=104) | p-value |
| --- | --- | --- | --- | --- | --- |
| Actual histopathology | Others | 86 (96.6) | 6 (40.0) | 92 (88.5) |  |
|  | Mucoepidermoid carcinoma, low grade | 3 (3.4) | 9 (60.0) | 12 (11.5) | < 1e-04 |

| **Statistics** | **Value** | **95% CI** |
| --- | --- | --- |
| Sensitivity | 60.00% | 32.29% to 83.66% |
| Specificity | 96.63% | 90.46% to 99.30% |
| Positive Predictive Value | 75.00% | 47.80% to 90.76% |
| Negative Predictive Value | 93.48% | 88.51% to 96.39% |
| Accuracy | 91.35% | 84.21% to 95.97% |

**Supplementary table 14: Prediction of Intermediate/high grade Mucoepidermoid carcinoma by Radiologist 3 using MRI signatures**

| Variable | Level | Others (n=88) | Mucoepidermoid carcinoma, intermediate/high grade (n=16) | Total (n=104) | p-value |
| --- | --- | --- | --- | --- | --- |
| Actual histopathology | Others | 84 (95.5) | 13 (81.2) | 97 (93.3) |  |
|  | Mucoepidermoid carcinoma, intermediate/high grade | 4 (4.5) | 3 (18.8) | 7 (6.7) | 0.1227 |

| Statistics | Value | 95% CI |
| --- | --- | --- |
| Sensitivity | 18.75% | 4.05% to 45.65% |
| Specificity | 95.45% | 88.77% to 98.75% |
| Positive Predictive Value | 42.86% | 15.62% to 75.24% |
| Negative Predictive Value | 86.60% | 83.56% to 89.14% |
| Accuracy | 83.65% | 75.12% to 90.18% |

**Supplementary table 15: Prediction of Mammary analogue secretory carcinoma histopathology by Radiologist 3 using MRI signatures**

| Variable | Level | Others (n=100) | Mammary analogue secretory carcinoma (n= 4) | Total (n=104) | p-value |
| --- | --- | --- | --- | --- | --- |
| Actual histopathology | Others | 97 (97.0) | 1 (25.0) | 98 (94.2) |  |
|  | Mammary analogue secretory carcinoma | 3 (3.0) | 3 (75.0) | 6 (5.8) | < 1e-04 |

| **Statistics** | **Value** | **95% CI** |
| --- | --- | --- |
| Sensitivity | 75.00% | 19.41% to 99.37% |
| Specificity | 97.00% | 91.48% to 99.38% |
| Positive Predictive Value | 50.00% | 22.27% to 77.73% |
| Negative Predictive Value | 98.98% | 94.67% to 99.81% |
| Accuracy | 96.15% | 90.44% to 98.94% |

**Supplementary table 16: Prediction of Carcinoma ex-pleomorphic adenoma histopathology by Radiologist 3 using MRI signatures**

| Variable | Level | Carcinoma ex-pleomorphic adenoma (n= 9) | Others (n=95) | Total (n=104) | p-value |
| --- | --- | --- | --- | --- | --- |
| Actual histopathology | Others | 6 (66.7) | 89 (93.7) | 95 (91.3) |  |
|  | Carcinoma ex-pleomorphic adenoma | 3 (33.3) | 6 (6.3) | 9 (8.7) | 0.03276 |

| **Statistics** | **Value** | **95% CI** |
| --- | --- | --- |
| Sensitivity | 33.33% | 7.49% to 70.07% |
| Specificity | 93.68% | 86.76% to 97.65% |
| Positive Predictive Value | 33.33% | 13.02% to 62.54% |
| Negative Predictive Value | 93.68% | 90.31% to 95.94% |
| Accuracy | 88.46% | 80.71% to 93.89% |

**Supplementary table 17: Prediction of Salivary duct carcinoma histopathology by Radiologist 3 using MRI signatures**

| Variable | Level | Others (n=87) | Salivary duct carcinoma (n=17) | Total (n=104) | p-value |
| --- | --- | --- | --- | --- | --- |
| Actual histopathology | Others | 81 (93.1) | 12 (70.6) | 93 (89.4) |  |
|  | Salivary duct carcinoma | 6 (6.9) | 5 (29.4) | 11 (10.6) | 0.01982 |

| **Statistics** | **Value** | **95% CI** |
| --- | --- | --- |
| Sensitivity | 29.41% | 10.31% to 55.96% |
| Specificity | 93.10% | 85.59% to 97.43% |
| Positive Predictive Value | 45.45% | 22.28% to 70.78% |
| Negative Predictive Value | 87.10% | 83.17% to 90.22% |
| Accuracy | 82.69% | 74.03% to 89.41% |

**Supplementary table 18: Prediction of Adenoid cystic carcinoma histopathology by Radiologist 3 using MRI signatures**

| Variable | Level | Others (n=100) | Adenoid cystic carcinoma (n= 4) | Total (n=104) | p-value |
| --- | --- | --- | --- | --- | --- |
| Actual histopathology | Adenoid cystic carcinoma | 4 (4.0) | 2 (50.0) | 6 (5.8) |  |
|  | Others | 96 (96.0) | 2 (50.0) | 98 (94.2) | 0.00551 |

| **Statistics** | **Value** | **95% CI** |
| --- | --- | --- |
| Sensitivity | 50.00% | 6.76% to 93.24% |
| Specificity | 96.00% | 90.07% to 98.90% |
| Positive Predictive Value | 33.33% | 11.25% to 66.35% |
| Negative Predictive Value | 97.96% | 94.74% to 99.22% |
| Accuracy | 94.23% | 87.87% to 97.85% |

**Supplementary table 19: Prediction of Non-Hodgkin lymphoma histopathology by Radiologist 3 using MRI signatures**

| Variable | Level | Others (n=101) | Non-Hodgkin lymphoma (n= 3) | Total (n=104) | p-value |
| --- | --- | --- | --- | --- | --- |
| Actual histopathology | Others | 100 (99.0) | 0 (0.0) | 100 (96.2) |  |
|  | Non-Hodgkin lymphoma | 1 (1.0) | 3 (100.0) | 4 (3.8) | < 1e-04 |

| **Statistics** | **Value** | **95% CI** |
| --- | --- | --- |
| Sensitivity | 100.00% | 29.24% to 100.00% |
| Specificity | 99.01% | 94.61% to 99.97% |
| Positive Predictive Value | 75.00% | 29.91% to 95.47% |
| Negative Predictive Value | 100.00% | 96.38% to 100.00% |
| Accuracy | 99.04% | 94.76% to 99.98% |
